# Perioperative, functional, and oncologic outcomes after ablation or partial nephrectomy for solitary renal tumors: a systematic review and meta-analysis of comparative trials

**DOI:** 10.3389/fonc.2023.1202587

**Published:** 2023-06-26

**Authors:** Zhi Wen, Li Wang, Jing Huang, Yang Liu, Cai-xia Chen, Chong-jian Wang, Lin-lin Chen, Xue-song Yang

**Affiliations:** ^1^ Department of Urology, Affiliated Hospital of North Sichuan Medical College, Nanchong, China; ^2^ Department of Hemodialysis, Sixth People’s Hospital of Nanchong, Nanchong, Sichuan, China

**Keywords:** solitary kidney, kidney cancer, ablation, nephrectomy, surgery

## Abstract

**Objectives:**

The perioperative, functional, and oncological outcomes of patients with solitary small renal tumors (SRMs) treated with ablation (AT) or partial nephrectomy (PN) remain controversial. The aim of this study was to compare the outcomes of these two surgical techniques.

**Methods:**

In April 2023, we conducted a literature search in several widely used databases worldwide, including PubMed, Embase, and Google Scholar. Review Manager was used to compare various parameters. The study was registered with PROSPERO (CRD42022377157).

**Results:**

Our final meta-analysis included 13 cohort studies with a total of 2,107 patients. Compared to partial nephrectomy (PN), ablation (AT) had shorter hospital stays (WMD -2.37 days, 95% CI -3.05 to -1.69; p < 0.00001), shorter operating times (WMD -57.06 min, 95% CI -88.92 to -25.19; p = 0.0004), less postoperative creatinine increases (WMD -0.17 mg/dL, 95% CI -0.29 to -0.05; p = 0.006), less postoperative glomerular filtration rate decreases (WMD -9.84 mL/min/1.73 m2, 95% CI -14.25 to -5.44; p < 0.0001), less postoperative new-onset chronic kidney disease (OR 0.33, 95% CI 0.16 to 0.71; p = 0.005), and less intraoperative blood loss (WMD -285.92 ml, 95% CI -428.44 to -143.40; p < 0.0001). The transfusion rate was lower in the ablation group (OR 0.17, 95% CI 0.06 to 0.51; p = 0.001). The risk of local recurrence was higher in the ablation group (OR 2.96, 95% CI 1.27 to 6.89; p = 0.01), while the risk of distant metastasis was higher in the partial nephrectomy group (OR 2.81, 95% CI 1.28 to 6.18; p = 0.01). The intraoperative and postoperative complication rates were lower in the ablation group (OR 0.23, 95% CI 0.08 to 0.62; p = 0.004 and OR 0.21, 95% CI 0.11 to 0.38; p < 0.00001, respectively). However, overall survival, postoperative dialysis rate, and tumor-specific survival were not different between the two groups.

**Conclusions:**

Our data suggest that ablation and partial nephrectomy are equally safe and effective in the treatment of small solitary kidney tumors and are better options for patients with poor preoperative physical condition or poor renal function.

## Introduction

Kidney cancer accounts for 2%-3% of all cancers, with an estimated hundreds of thousands of new cases and tens of thousands of deaths worldwide each year (1). It is generally detected incidentally on non-invasive imaging, and most incidentally detected renal masses are usually small, with small renal masses (SRMs) measuring less than 4 cm (stage T1a) accounting for about half of all renal cancers (2). With technological advances, the surgical approach to renal cancer has evolved from a single previous open nephrectomy to multiple surgical options today, including partial nephrectomy, radiofrequency ablation, and cryoablation (3). The European Association of Urology (EAU) refers to recommending partial nephrectomy as the standard surgical approach for small renal masses. However, small renal tumors of the solitary kidney are still a challenging treatment problem for urologists, and the choice of surgical approach remains controversial. For solitary renal tumors, renal function preservation must be considered because the patient has only one kidney. While partial nephrectomy, due to renal ischemia, can have a greater impact on renal function, patients with significant loss of renal function may develop chronic kidney disease (CKD), leading to increased risk of acute kidney injury (AKI) and mortality (4). Additionally, partial nephrectomy has its own technical challenges, such as high complication rates and serious complications, such as blood loss and urinary leakage that require transfusion (5). Ablation (AT) techniques involve using different energies to achieve tumor mass necrosis (6). Minimally invasive treatments, which utilize heat- or cold-based energies, are of concern because they better preserve overall renal function (7). The most widely used ablation therapies include radiofrequency ablation (RFA) and cryoablation (CA) (8). The safety and efficacy of ablation techniques for solitary renal tumors were reported as early as 2003 (9). AT can be a good alternative to PN, particularly in patients with small solitary kidney tumors. This is because warm ischemia does not cause renal dysfunction, it maximally preserves renal function, reduces the risk of postoperative renal loss, and is less technically challenging. It does not require incision of the renal parenchyma, and it is more minimally invasive, with fewer complications (10). Recent literature reports that several study groups have reported favorable outcomes with radiofrequency ablation of renal masses with 2-5 year follow-up (11–13). Mid-term follow-up results of patients treated with ablative techniques showed good local tumor control (14). Cryoablation has also been documented to have fewer technical challenges, favorable oncologic outcomes, and shorter hospital stay (15). Ablation (radiofrequency and cryoablation) is increasingly used in elderly patients and high-risk surgical patients. The guidelines consider AT as an alternative to PN in patients who are not candidates for surgery, particularly in patients with severe comorbidities who are not candidates for surgery, and in patients with urgent indications for nephron-sparing surgery (16).

In recent years, numerous studies have been published attempting to explore whether TA is an effective and safe alternative for the treatment of SRMs in solitary kidneys. However, the results have been inconsistent (17–29). Additionally, the sample size at single centers was small, and no credible conclusions could be drawn. Therefore, this systematic review and meta-analysis compared perioperative, functional, and oncologic outcomes between the two surgical modalities.

### Literature search

This study adhered to the standards specified in PRISMA (30)((Preferred Reporting Items for Systematic Reviews and Meta-Analysis) and was prospectively registered in the PROSPERO database (CRD42023 411427). Articles included in the systematic review were investigated independently by two reviewers (WZ and WL). The data obtained from the literature were before April 1, 2023. Extensive literature searches were conducted using MEDLINE, EMBASE, and Google Scholar databases. The search was limited to English language papers. Medical Subject Headings (Mesh) terms and keywords, such as “ Solitary Kidney*”, “ Ablation*” OR “Cryoablation*”, “ Partial Nephrectomy”. In addition, we manually searched and reviewed relevant references to avoid any omission.

### Selection criteria

The inclusion and exclusion criteria for the studies were determined using the PICOS method. The inclusion criteria consisted of solitary renal tumors undergoing ablation in the experimental group and partial nephrectomy in the control group. The primary outcome measures of this study were changes in creatinine and estimated glomerular filtration rate, new onset of chronic kidney disease after surgery, postoperative dialysis rate (percentage of patients requiring dialysis after treatment), local recurrence, and postoperative metastasis. Secondary outcome measures included length of hospital stay, operation time, complications (intraoperative complications, postoperative complications, major complications), intraoperative blood loss, blood transfusion, overall survival, and cancer-specific survival. Cohort studies, case-control studies, and randomized controlled trials (RCT) were eligible for inclusion. Non-comparative studies, editorial comments, conference abstracts, case reports, unpublished studies or comments, articles published in non-English language, and studies with no data available were excluded. Data extraction was performed independently by two reviewers and included general information such as the first author, publication year, and demographic characteristics such as age, sex, follow-up time, tumor size, and perioperative outcomes such as operative time, length of hospital stay, and intraoperative complications.

### Study screening and selection

Two independent authors (WZ and WL) manually screened all retrieved records. When consensus could not be reached between the two authors, it was resolved by consultation with a third author (LY). Retrospective, prospective, non-randomized, and randomized studies were included according to PICOS criteria. Reviews, conference abstracts, case reports, letters to editors, and editorials were excluded. Papers were selected for screening by reading the full text if found to be relevant to the objectives of this study.

### Statistical analysis

This study used Review Manager V5.4.1 software (Cochrane Collaboration, Oxford, UK) for statistical analysis. Results are presented as 95% confidence intervals (CI) and odds ratios (OR) for dichotomous variables and weighted mean difference (WMD) for continuous variables. Data from some studies reporting only medians, quartiles, or extreme value ranges were converted to means and standard deviations (SDs) using data conversion tables provided by McGrath (31). Meta-analysis was performed using the Mantel-Haenszel method for dichotomous variables and the inverse variance method for continuous variables. For survival data, because some articles do not give survival curves, we analyze survival data in the form of binary variables, the simplest way is to collect them directly from the original article, and if the data are provided only in the form of survival curves, we extract survival rates from some designated times. We used a random-effects model for all analyses due to the predictable significance of heterogeneity across trials. Study heterogeneity was calculated using the I (2) statistic, with 0 – 40% defined as mild heterogeneity; 40% – 60% as moderate heterogeneity; 60 – 75% as large heterogeneity; and 75 – 100% as high heterogeneity (32). Values of p < 0.05 were considered statistically significant.

### Bias risk assessment

We included articles that were all cohort studies and no randomized cohort studies were identified. The risk of bias was assessed using the Newcastle-Ottawa Scale (NOS) and the quality of the literaturetative evaluation was evaluated using a semiquantistar system, which consisted of 9 stars.

### Sensitivity analysis

We used the leave-one-out method to exclude studies from the pooled effect one at a time to assess the robustness of the estimates. Furthermore, we evaluated the robustness based on the study cohort size (excluding studies with < 100 patients), which may contribute to heterogeneity. However, we cannot perform sensitivity analyses comparing three or fewer studies.

### Publication bias

Funnel plots were used to screen for potential publication bias.

## Result

### Baseline characteristics

**Figure 1:**
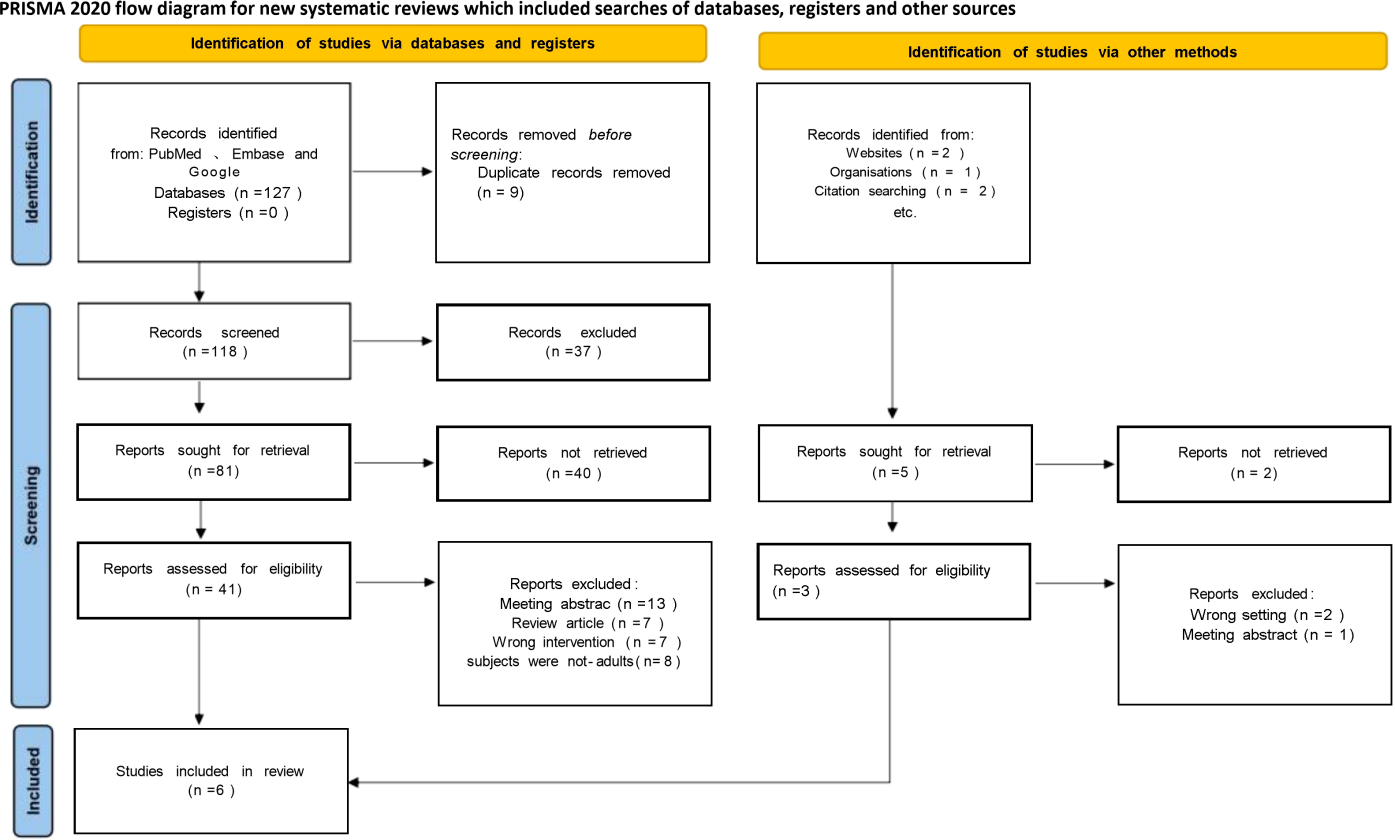
PRISMA flowchart.

The initial literature search retrieved 53 articles. After removing nine duplicates, 44 studies remained for screening. Fifteen of these papers were excluded from title and abstract screening as they were not relevant to the objectives of this study. The full texts of the remaining 29 studies were screened, and 16 papers lacking data specificity, wrong interventions, etc., were further excluded. Finally, 13 studies involving 2107 patients were accepted and included in the meta-analysis. **Table 1** summarizes the baseline characteristics and preoperative variables of the included patients (sample size, age, body mass index (BMI), preoperative creatinine, preoperative glomerular filtration rate, preoperative follow-up time, number of patients with preoperative chronic kidney disease, tumor size, preoperative American Society of Anesthesiologists (ASA) score), RENAL nephrometry score (33). **Table 2** summarizes the surgical, complication, functional, and oncologic findings, and it can be seen from **Table 3** that the relevant characteristics and variables of this study are comparable. There were no significant differences between the groups in age (P = 0.09), male sex (P = 0.16), BMI (P = 0.57), and follow-up time (P = 0.79), but there were some differences in baseline tumor size (P = 0.002), indicating that tumors were smaller in the ablation (AT) group than in the partial nephrectomy (PN) group. The same preoperative creatinine baseline (P < 0.00001) showed some differences, indicating worse preoperative renal function in the AT group. **Table 4** summarizes the overall and cancer-specific survival of the included patients. **Figure 1** shows the flow diagram of the PRISMA process.

**Table 1:** Baselines characteristics of included studies.

Reference	§	Type	N*	Age	Male	BMI kg/m^2^)	Preoperative renal function			Pathology	Tumor Size(cm)	ASA	RENAL nephrometry score	Follow-up^#^
							Creatinine	eGFR	CKD(%)	Be/Ma/Un				
Beksac 2022	I	CA	43	68 (61, 73)	31	28 (26, 33)	NA	56 (45, 66)	30 (70%)	10/23/10	2.00 (0.80-3.8)	34 (83%)	7.0 (5.75, 8.00)	70 (43-112)
		PN	31	59 (56, 66)	17	31 (28, 35)	NA	58 (46, 74)	16 (52%)	3/28/0	2.80, (1.3-4.0)	25 (83%)	6.00 (5.00, 9.50)	40 (15-61)
Bhindi 2018	I	CA	54	65 (54–75)	39	NA	NA	56 (48–69)	NA	NA	3.5 (2.3–6.5)	NA	7 (6–9)	NA
		PN	64	63 (58–67)	47	NA	NA	56 (47–77)	NA	NA	3.7 (2.0–5.0)	NA	8 (5–9)	NA
Goyal 2011	R	CA	23	68.4 (39.8-79.4)	13	NA	1.3±0.4	54.6±16.5	15 (65%)	5/18/0	2.5 (1-4)	NA	NA	31.2 (0.6-153)
		PN	15	65.2 (47.2-85.3)	13	NA	1.5±0.7	55.07±22.2	9 (60%)	3/12/0	3.4 (1-5.5)	NA	NA	30.8 (0.1-113.5)
Haber 2012		CA	30	60.9 ± 11.4	22	31.5 ± 5.8	1.5 ± 0.5	53.8 ± 19.0	NA	5/25/0	2.6 ± 1.08	NA	NA	60.2 ± 46.3
		LPN	48	60.6 ± 13.7	25	30.1 ± 6.2	1.2 ± 0.3	61.6 ± 18.6	NA	12/36/0	3.2 ± 1.33	NA	NA	42.7 ± 30.8
Mitchell 2011	R	AT	50	63 (27–83)	33	NA	1.3 (0.8–2.6)	53.3±12.3	34 (68%)	NA	2.5 (1.2–7.3)	38 (76%)	NA	NA
		PN	62	69 (49–89)	49	NA	1.5 (0.7–3.3)	56.2±22.3	44 (72%)	NA	3.5 (0.7–13.0)	39 (63%)	NA	NA
Mues 2012	R	AT	98	64 (38–86)	64	NA	1.4	59		6/69/23	2.5 (1–4.4)	NA	NA	31
		PN	100	64 (35–92)	64	NA	1.4	59	NA	97/3/0	3.9 (1–10)	NA	NA	24
Olweny 2012	I	PFA	37	63.8 (56.3–69.1)	24	NA	NA	NA	NA	37/0/0	2.1 (1.8–2.8)	NA	NA	6.5 (5.8–7.1)
		PN	37	54.8 (47.8–59.1)	20	NA	NA	NA	NA	37/0/0	2.5 (1.7–3.1)	NA	NA	6.1 (5.4–7.3)
Pandolfo 2022	I	AT	119	66.1 ±12.6	NA	29.6 ±6.1	1.43 ±0.53	53.7 ±17.4	79 (66%)	NA	2.5 ±1.3	81 (66%)	5 (3)	46 ±39.4
		RAPN	50	65.5±9.0	NA	26.6±4.2	1.2 ±0.32	63.9 ±24.6	28 (56%)	NA	2.8 ±1.1	22 (44%)	7 (2)	37 ±30
Panuma 2013	I	CA	43	64 (57-72)	35	29 (26-32)	1.3 (1.2-1.5)	57 ±9.2	30 (70%)	13/25/5	2.2 (1.6-3.2)	36 (83%)	NA	41 (26-59)
		PN	33	60 (51-69)	22	29.1 (26-32)	1.2 (1-1.4)	62±17.	14 (42%)	9/24/0	2.9 (1.8-4.2)	17 (59%)	NA	17 (5-62)
Raman 2010	I	PFA	47	65.9 (16.7)	33	NA	NA	46.5 (15.9–91.6)	40 (85%)	5/40/8	2.7 (1.5–6.5)	NA	NA	18.1 (6–66)
		OPN	42	59.6 (12.8)	26	NA	NA	55.9 (30.5–89.7)	22 (52%)	9/37/0	3.5 (1.3–5.5)	NA	NA	30.0 (11–83)
Turna 2009	R	RFA^a^	29	60.7±14.3	18	30.0 ± 7.5	1.4 ± 0.5	53.2 ± 16.2	17 (59%)	5/24/0	2.6 ± 1.0	20 (69%)	NA	14.0 (1–44)
		CA^b^	36	64.1±11.1	23	31.3 ± 5.7	1.4 ± 0.5	52.3 ± 19.7	26 (72%)	8/22/6	2.5 ± 1.1	28 (78%)	NA	24.0 (1–84)
		LPN	36	60.3±15.5	21	30.5 ± 7.1	1.2 ± 0.4	65 ± 23.5	23 (64%)	12/23/1	3.7 ± 1.9	24 (67%)	NA	42.5 (7-81)
Xiaobing 2017	R	RFA	16	59.6 (36–79)	11	NA	94.9 (66–113)	NA	NA	0/14/2	3.4 (1.5–6.0)	NA	NA	33.7 ± 22.9
		PN	20	62.1 (41–83)	15	NA	91.4 (53–174)	NA	NA	2/18/0	3.6 (1.5–6.0)	NA	NA	26.1 ± 16.2
Yasuda 2022	I	CA	102	67 (58–73)	69	31 (27–35)	NA	53 (40–65)	NA	NA	2.3 (1.6–3.1)	NA	6 (4–8)	31.2 (6.8–114.9)
		PN	842	64 (55–70)	535	29 (25–33)	NA	57 (44–71)	NA	NA	3.9 (2.8–5.3)	NA	9 (7–10)	57.4 (47.2–71.1)

CA, cryoablation; AT, ablation therapy; RFA, radiofrequency ablation; PN, partial nephrectomy; RAPN, robot-assisted partial nephrectomy; OPN, open partial nephrectomy; LPN, laparoscopic partial nephrectomy; ASA, Percent of patients with American society of anesthesiologists (ASA) score 3 or 4; CKD, chronic kidney disease; BMI, body mass index; GFR, glomerular filtration rate; NA, not applicable; CKD:GFR<60 ml/min/1.73m2;M(SD); Be/Ma/Un = benigh/malignant/unknown; ^#^ Follow up duration in months; ^§^ Data a (b-c) are presented as quartiles or extreme values and d ± e as mean ± standard deviation in the table; N* Number of Patients; Type: Surgical method; I, interquartile range; R, Rang.

**Table 2:** Perioperative outcomes.

Reference	Type	N*	hospital stay	operating time	Post operative SCr (mg/dL)	SCr increase (mg/dL)	Post operative eGFR (mL/min/1.73m2)	Percent eGFR decrease after surgery (%)	New onset CKD	Blood loss in cc	T	D	L	Me	I	P	M
Beksac 2022	CA	43	NA						4	NA		0	11	6	NA	NA	1
	PN	31							6			1	1	1		NA	2
Bhindi 2018	CA	54							NA			NA	NA	NA		NA	NA
	PN	64							NA			NA	NA	NA		NA	
Goyal 2011	CA	23	2.6±2.0	NA	1.33±0.4	NA	51.9±16.5	4.7±15.9	2	87±105.7	0	1	1	2		2	
	PN	15	6.7±3.7	NA	1.9±0.7	NA	43.5±18	17.7±26.7	2	316±528.3	1	2	2	1		8	
Haber 2012	CA	30	2.4 ± 2.2	197.4 ± 52.6	1.7 ± 0.6	0.2 ± 0.3	47.5 ± 14.8	11.0 ± 16.1	NA	162.4 ± 163.2	1	0	4	4	1	2	
	LPN	48	4.6 ± 2.9	227.7 ± 73.1	1.7 ± 0.9	0.4 ± 0.5	47.5 ± 18.4	21.4 ± 21.9	NA	391.3 ± 692.0	8	3	0	1	4	11	
Mitchell 2011	AT	50	NA		NA	0.1±0.4	50.6±16.5	3.4±18	2	NA	NA	0	NA				
	PN	62			NA	0.1±0.6	51.9±19.4	7±25	5	NA	1	NA					
Mues 2012	AT	98	2.0±1.4	158.3±46.3	NA				NA	67.3±79.2	0		7	2	1	9	4
	PN	100	4.8±4.0	169±40.4					NA	575.4±775.4	4		3	1	6	18	8
Olweny 2012	PFA	37	NA						NA	NA	NA		3	1	NA		
	PN	37							NA	NA			3	3			
Pandolfo 2022	AT	119	2.7 ±3.2	83 ±75					NA	NA			NA	NA	2	6	1
	RAPN	50	4.7 ±2	200±92					NA	NA			NA	NA	2	5	1
Panuma 2013	CA	43	1 ±0	178.1 ± 51.3					3	60.9±23.7	1	2	12	6	0	3	1
	PN	33	4 ±1.5	238.2±96.8					8	250 ±232.4	7	0	0	2	5	13	6
Raman 2010	PFA	47	NA		NA			10.4±16	0	NA	NA	2	5	NA	NA	NA	NA
	OPN	42						24.5±26	7	NA	NA	1	3	NA		NA	
Turna 2009	RFA^a^	29	NA	NA	1.5 ± 0.8	0.1 ± 0.4	52 ± 15	13.2±28.3	NA	NA	1	0	13	4		2	
	CA^b^	36	1.8 ± 1.3	181 ± 69	1.6 ± 0.7	0.2 ± 0.3	51 ± 27		NA	151 ± 171		0	6	3		5	
	LPN	36	3.3 ± 2.6	223 ± 81	1.6 ± 0.6	0.4 ± 0.5	48 ± 17	27.7±27.7	NA	408 ± 800	0	3	0	0		21	
Xiaobing 2017	RFA	16	13.6±6.2	148.3±59.3	NA						NA	NA	1	1	1	2	
	PN	20	19.5±8.8	214.7±101.6							NA		3	0	5	4	
Yasuda 2022	CA	102	NA	173.3±63.1							0		7	NA	NA	NA	6
	PN	842	NA	250.8±68.3							53		31	NA	NA	NA	69

I, Intra-operative complication; P, Post-operative complication; M, Major complications; T, Transfusion; L, Local recurrence; D, Dialysis; Me, Metastasis; *N, number; NA, Not Available.

**Table 3:** The demographics of the studies.

Variable	Number of studies with available data	WMD/OR	95% CI	p value
Age (years)	14	1.96	--4.58	0.09
BMI (kg/m2)	6	0.41	- 1- 1.82	0.57
Male (*n*)	13	1.21	0.93-1.59	0.16
Stone size (mm)	14	--0.65	-1.07-0.23	0.002
GFR	1 1	--5.59	-7.79--3.4	0.0001
follow-up	1 1	1.19	-7.73-10.12	0.79

WMD, weighted mean diference; OR odds ratio; Cl, confdence interval.

**Table 4:** The overall and cancer-specific survival.

Reference	Follow-up number	Overall survival(year)			cancer-specific survival(year)		
		1	3	5	1	3	5
Beksac 2022	33	32	26	NA	NA		
	28	25	17	NA			
Goyal 2011	23	21	17	11			
	15	13	12	12			
Haber 2012	30	30	28	26	NA	28	28
	48	44	44	44	NA	44	44
Olweny 2012	37	36	33	33	36	33	33
	37	37	37	34	37	37	34
Turna 2009	24	24	16	NA	23	16	NA
	22	21	16	NA	21	16	
	23	21	16	NA	21	16	

NA, Not Available.

### Assessment of quality

All articles were cohort studies, and therefore the Newcastle-Ottawa Scale (NOS) was used for quality evaluation of the literature. Studies defined literature NOS ≥ 6 stars as high-quality literature. A total of 12 of the 13 studies were of high quality, but Raman provided experiments that were of low quality. **Table 5** shows the details of the quality assessment of the cohort studies.

**Table 5:** Study quality of case-control studies based on the NWewcastle-Ottawa scale.

NOS	Selection				Comparability	Outcome			Overall score
**ID**	**1**	**2**	**3**	**4**	**5**	**6**	**7**	**8**	
Beksac 2022	★	★	★	★	★	★	★	★	8
Bhindi 2018	★	★	★	★	★	★	★	★	8
Goyal 2011		★	★	★	★	★	★	★	7
Haber 2012		★	★	★	★	★	★	★	7
Mitchell 2011		★	★	★	★	★	★	★	7
Mues 2012		★	★	★	★	★	★	★	7
Olweny 2012		★		★	★	★	★	★	6
Pandolfo 2022	★	★	★	★	★	★	★	★	8
Panumatrassamee 2013		★		★	★	★	★	★	6
Raman 2010		★		★	★		★	★	5
Turna 2009		★		★	★	★	★	★	6
Xiaobing 2017	★	★	★	★	★	★	★	★	8
Yasuda 2022	★	★	★	★	★	★	★	★	8

1, Representativeness of the exposed cohor; 2, Selection of the nonexposed cohort; 3, Assessment of exposure; 4, Demonstration that outcome of interest was not present at start of study; 5, Comparability of cohorts on the basis of the design or analysis; 6, Ascertainment of outcome ; 7, Long enough follow-up for outcomes to occur; 8, Adequacy of follow-up of cohorts. ★, A star represents a point.

### Meta-analysis of perioperative outcomes

**Figure 2:**
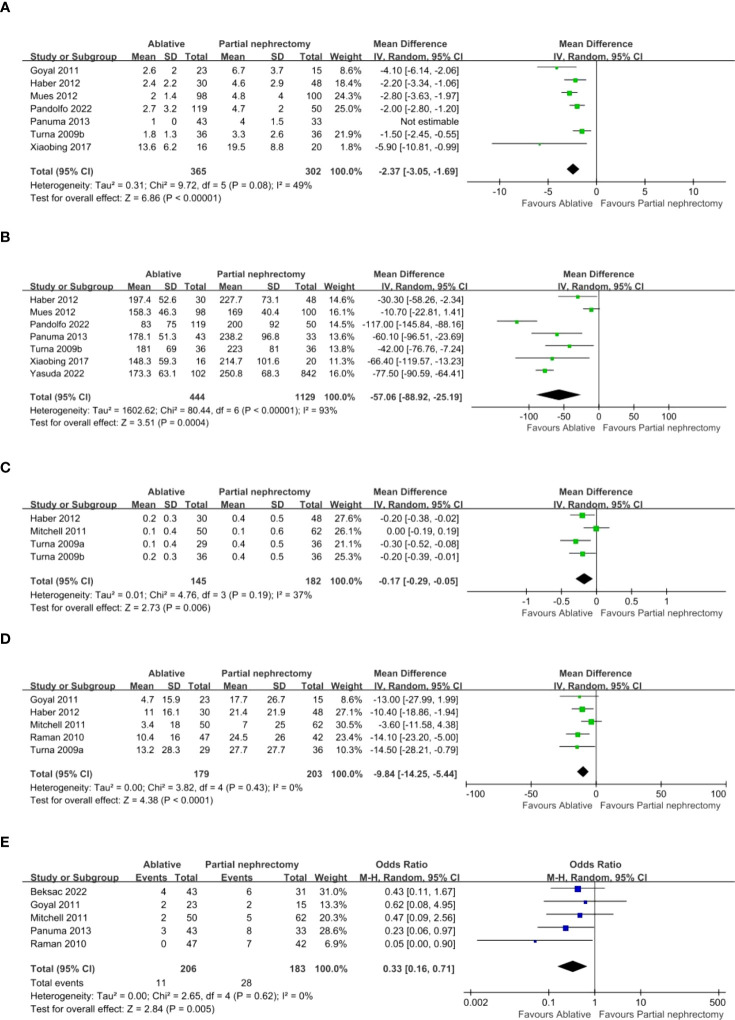
**(A)** length of hospital stay **(B)** operative time **(C)** post operative SCr increase **(D)** Percent eGFR decrease after surgery **(E)** New onset CKD.

**Figure 3:**
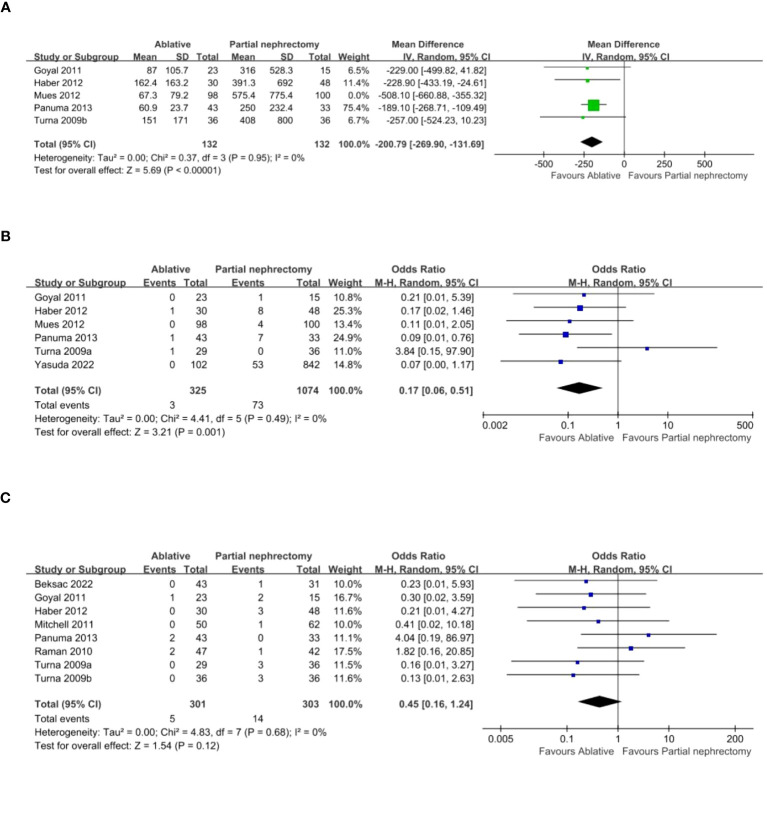
**(A)** estimated blood loss **(B)** transfusion rates **(C)** Dialysis.

There were 13 studies, but Turna provided data that could be divided into radiofrequency ablation (RFA) and cryoablation (CA) as well as partial nephrectomy (PN) for comparison, which we labeled Turna a and Turna b. Seven studies provided details of hospital stay and operation time. Compared with the PN group, the AT group (ablation group) had shorter hospital stay (WMD -2.37 day, 95% CI -3.05, -1.69; p < 0.00001) (**Figure 2A**), shorter operation time (WMD -57.06 min, 95% CI -88.92, -25.19; p = 0.0004) (**Figure 2B**). Four studies provided changes in postoperative creatinine, and the AT group had less postoperative creatinine increase (WMD -0.17 mg/dL, 95% CI -0.29, -0.05; p = 0.006) (**Figure 2C**). Pooled analysis of 5 studies showed that the AT group had less postoperative glomerular filtration rate decrease (WMD -9.84 mL/min/1.73 m2, 95% CI -14.25, -5.44; p < 0.0001) (**Figure 2D**) and less postoperative new chronic kidney disease (OR 0.33, 95% CI 0.16, 0.71; p = 0.005) (**Figure 2E**). intraoperative blood loss was less (WMD -285.92 ml, 95% CI -428.44, -143.40; p < 0.0001) (**Figure 3A**). Pooled analysis of six studies showed that the AT was associated with a lower transfusion rate relative to the PN (OR 0.17, 95% CI 0.06, 0.51; p = 0.001) (**Figure 3B**). However, there was no statistical difference in the postoperative dialysis rate between the two surgical methods (OR 0.45, 95% CI 0.16, 1.24; p = 0.12) (**Figure 3C**).

### Meta-analysis of complications

**Figure 4:**
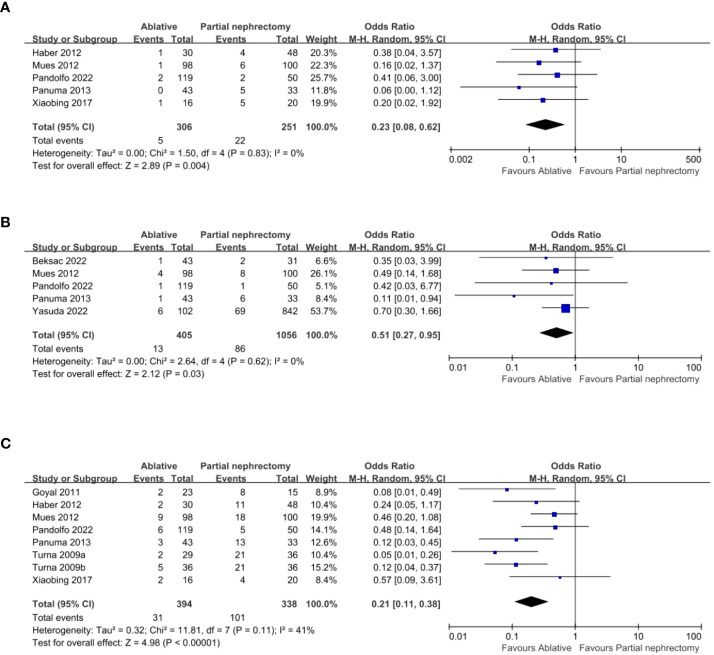
**(A)** Intra-operative complication **(B)** Major complications **(C)** Post-operative complication.

Five studies provided data on intraoperative complications, which were summarized and showed that the AT group had fewer intraoperative complications (OR 0.23, 95% CI 0.08-0.62; p = 0.004) (**Figure 4A**) and fewer major complications (OR 0.51, 95% CI 0.27-0.95; p = 0.03) (**Figure 4B**) than the PN group. Eight studies were pooled, and it showed that postoperative complications were less frequent in the AT group (OR 0.21, 95% CI 0.11-0.38; p < 0.00001) (**Figure 4C**).

### Meta-analysis of functional outcomes

**Figure 5:**
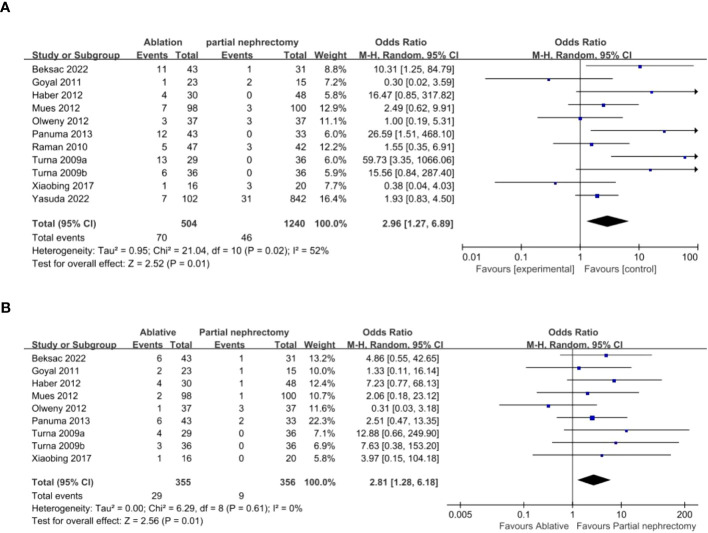
**(A)** Local recurrence **(B)** metastasis.

**Figure 6:**
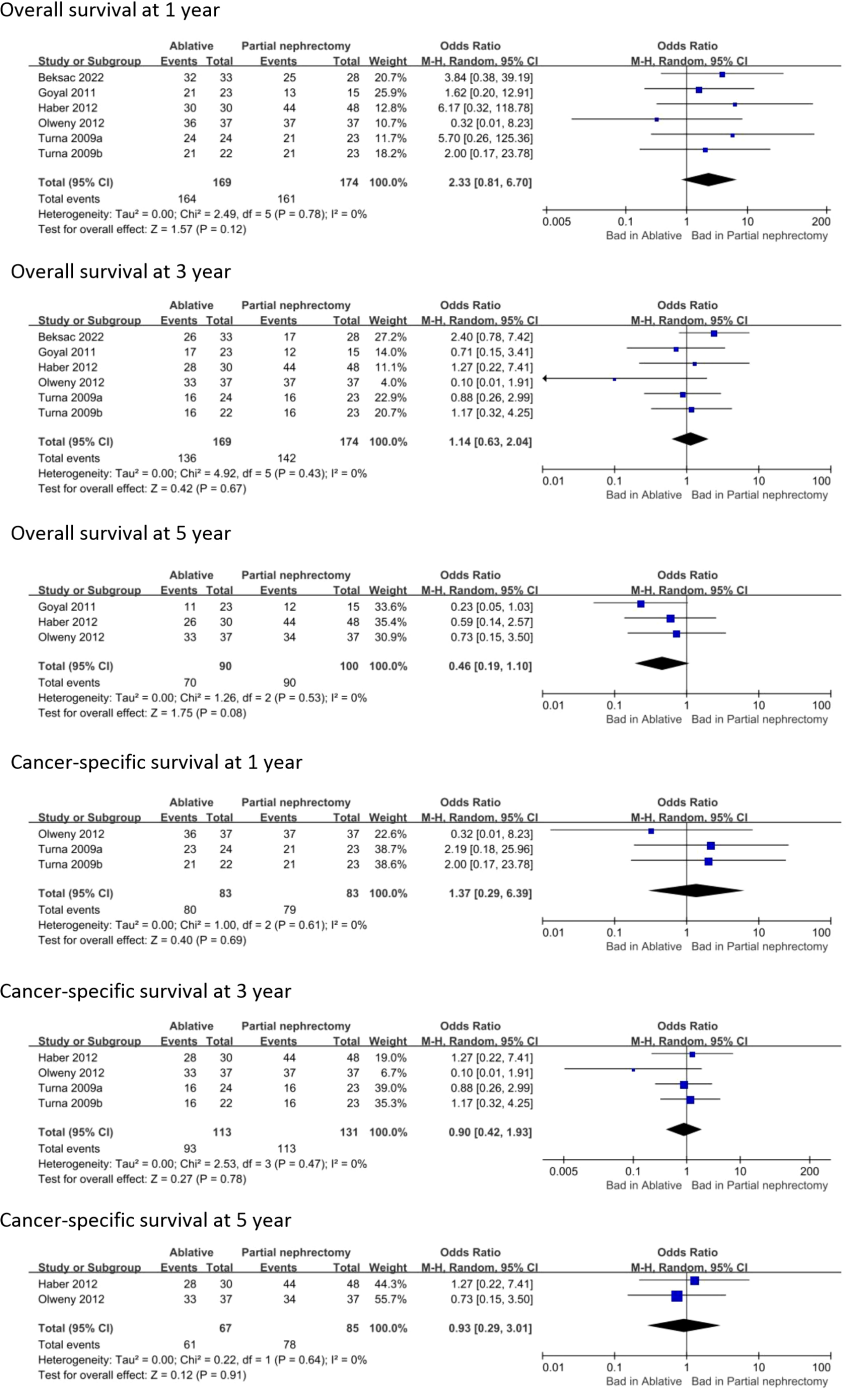
Overall survival and Cancer-specific survival.

Data analysis of 11 studies showed a higher risk of local recurrence in the AT group compared to the PN group (OR 2.96, 95% CI 1.27-6.89; p = 0.01) (**Figure 5A**). Additionally, analysis of 9 studies revealed a higher risk of distant metastasis in the AT group (OR 2.81, 95% CI 1.28-6.18; p = 0.01) (**Figure 5B**). However, there was no significant difference in overall survival or cancer-specific survival between the two surgical modalities (**Figure 6**).

### Sensibility analysis

**Figure 7:**
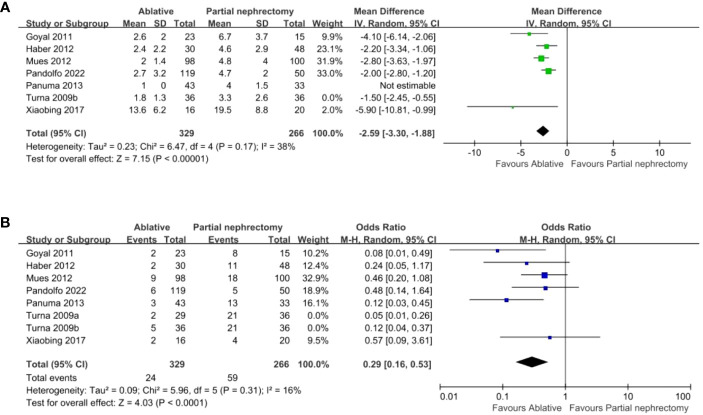
**(A)** length of hospital stay **(B)** Post-operative complication.

In this meta-analysis, some results showed significant heterogeneity (I2 = 49% for hospital stay, I^2 = ^93% for operation time, I^2 = ^37% for postoperative creatinine increase, I^2 = ^70% for blood loss, I^2 = ^52% for local recurrence rate, and I^2 = ^41% for postoperative complications). The heterogeneity of operation time and blood loss was high, and hospital stay, postoperative creatinine increase, local recurrence rate, and postoperative complications had some heterogeneity. The other results showed no significant heterogeneity. We performed sensitivity analyses on target parameters to obtain stable, convincing conclusions. We excluded studies using a leave-one-out approach from the pooled effect and found that heterogeneity decreased from I^2 = ^49% to 38% after removing data provided by Turn 2009b. This may be because patients provided by Turn 2009b often received sedative analgesia in outpatients and percutaneous fine-needle ablation under CT guidance, which greatly reduced length of stay. This may also explain the reduction in heterogeneity from the original I^2 = ^41% to I^2 = ^16% after removing the two sets of data provided by Turn 2000 for postoperative complications. Because more minimally invasive procedures seem to be associated with fewer complications. After removing Turn 2009 data using a leave-one-out approach for length of hospital stay and postoperative complications, the results still suggest a potential advantage for AT over PN in terms of operative time and intraoperative complications. The remaining results were found to be stable after exclusion using the leave-one-out method (**Figure 7**).

## Discussion

### Perioperative outcomes

The perioperative parameters that varied most significantly between the AT and PN groups were length of hospital stay, operation time, postoperative creatinine change, postoperative glomerular filtration rate change, estimated blood loss, postoperative new chronic kidney disease rate, blood transfusion, and postoperative dialysis rate. However, except for postoperative dialysis rate, the remaining perioperative parameters suggested that AT had an advantage over PN. Compared to partial nephrectomy, percutaneous ablation is more commonly used in the ablation group. In the Panumatrassame study, patients were assigned to receive percutaneous or laparoscopic ablation based on tumor location and technical ability at that time. Percutaneous ablation resulted in a significant reduction in operative time, and even partial ablation procedures could be performed as outpatients (27). Aspercutaneous ablation is more minimally invasive than partial nephrectomy, the advantages in terms of blood loss and transfusion rate are predictable.

In the Mues study, 98 patients underwent ablation surgery, and intraoperative blood loss was significantly reduced compared with 100 patients who underwent partial nephrectomy. None of the ablation patients received blood transfusion, and the amount of intraoperative blood loss was partly related to the rate of blood transfusion, which was also confirmed by relevant reports (34). Additionally, patients in the included literature tended to have larger tumors in the partial nephrectomy group compared to the ablation group, which means more difficult procedures and bleeding. A pooled analysis of 14 studies in 13 articles showed that renal ablation has a smaller impact on renal function relative to partial nephrectomy, as demonstrated by less creatinine increase and glomerular filtration rate decrease after renal ablation. This may be explained by the greater surgical trauma of PN than RFA, which can aggravate the damage to the kidney. Studies have shown that partial nephrectomy often requires clamping the renal artery, which can lead to warm ischemia of the patient ‘s kidney, resulting in irreversible renal impairment (35). In addition, PN may remove more renal parenchyma, which will to some extent affect postoperative renal function more. For patients with solitary renal tumors, the quality of renal function is often very important because a lonely kidney is the most important risk factor for postoperative acute renal failure, and increased risk of worsening renal function is associated with cardiovascular morbidity and mortality (36). Preoperative conditions can influence changes in creatinine and glomerular filtration rate after nephrectomy. Analysis of the baseline data of the included articles found that patients with solitary kidney tumors who chose radiofrequency ablation tended to have lower preoperative glomerular filtration rates. Although analysis of the included articles found no statistical difference in age between the two surgical modalities, in-depth analysis of the various studies found that the radiofrequency group tended to be older, with a mean age of 64 years in the ablation group and 60 years in the partial nephrectomy group, as shown in the Panuma 2013 study. Walach (37) et al. recently showed that factors such as poor preoperative patient condition, older age, and solitary kidney tumor were decisive factors in allowing clinicians to choose ablation over other procedures. In other words, because of factors such as poor preoperative physical condition or poor preoperative renal function, only more minimally invasive modalities and treatments that have less impact on renal function are selected for this patient. This inevitably leads to better perioperative outcomes.

### Complications

Regarding the definition of complications, because the information in the literature was incomplete, we only collected and analyzed the information related to intraoperative complications, postoperative complications, and major complications according to the Clavien-Dindo classification (Clavien score ≥ 3) (38). A pooled analysis of the data from the included studies showed that ablation was superior to partial nephrectomy in terms of intraoperative, postoperative, and major complications. This is consistent with the conclusions of Larcher (34) and Pierorazio (39). Compared with partial nephrectomy, ablation has the advantages of minimal invasiveness and faster postoperative recovery. In the Panuma 2013 study, ablation was performed in 43 patients, and postoperative complications occurred in only 3 patients compared to 13 patients in the PN group. However, with the development of surgical robots, robotic surgery has better vision, more flexible and precise operations, and robot-assisted laparoscopic partial nephrectomy can reduce the occurrence of complications to some extent compared with traditional laparoscopic and open surgery (40). As shown in the Pandolfo 2022 study, there was no significant difference in complications between the PAPN and AT groups.

### Oncologic outcomes and follow-up

A pooled analysis of the data included in the study showed that the risk of local recurrence and the risk of distant metastasis were higher in the ablation group compared with the partial nephrectomy group. The main goal of treating solitary renal tumors is not only to maintain better renal function but also to achieve better tumor control. However, this does not mean that the ablation procedure cannot be selected for patients. Unlike partial nephrectomy, ablation can be performed multiple times without increasing the corresponding difficulty of the procedure (7). In the Xiaobing study, 1 of 16 ablated patients showed recurrence after surgery, and the patient underwent ablation surgery again 8 months later, and no recurrence was found during long-term follow-up. A pooled analysis showed no statistical difference in overall survival and tumor-specific survival between the two treatment modalities. Bianchi (41) et al. reported similar overall survival between the radiofrequency ablation and partial nephrectomy groups (91% vs 95.8%, P = 0.6). These results imply that although ablation is associated with a higher risk of local recurrence and distant metastasis than PN, no significant difference in overall survival is observed between the two treatment modalities. Unfortunately, most of the data we included in the study had only 5 years of follow-up. This conclusion needs to be confirmed by more prospective randomized trials with long-term rigorous follow-up. The duration of follow-up and the items examined at follow-up were not nearly the same in each study. For example, in Haber’s study, postoperative follow-up of patients who underwent partial nephrectomy included abdominal CT or MRI at 6 months, and imaging studies were performed annually thereafter for patients with pathologically confirmed renal cancer. In patients undergoing LCA, CT-guided percutaneous biopsy was performed 6 months after surgery. In Mitchell’s study, all patients underwent imaging at 3 months after surgery. Further follow-up was at the discretion of the treating physician. Almost all patients underwent regular renal function tests after surgery, but changes in renal function after surgery have not been further investigated because of insufficient data. Longer follow-up allows better understanding of oncologic findings and changes in renal function. Therefore, the AUA recommends at least 5 years of follow-up to observe late recurrence (42).

## Limitations

This study has several limitations that need to be considered. First, no further subgroup analysis was performed because fewer articles were included for radiofrequency ablation and cryoablation. Second, analysis of the baseline data included in the study showed that patients in the AT group tended to be older, have smaller tumors, and have worse preoperative renal function, which may overestimate the advantages of AT in terms of complications, perioperative outcomes, etc. Finally, the study included is based on cohort studies, and further larger randomized trials are needed to provide more reliable evidence for the results of the pooled analysis.

## Conclusion

Both partial nephrectomy and ablation are safe and effective treatments for small solitary kidney tumors. Ablative therapy is associated with shorter procedure times, fewer complications, and less renal impairment than partial nephrectomy. Although ablation is relative to a greater risk of local recurrence and metastasis compared to partial nephrectomy, there is no apparent difference in overall and cancer-specific survival. Ablation is a better option for patients with poor physical condition or preoperative renal function.

## Author contributions

Substantial contributions to the concept or design of the work, X-SY. Data extraction and literature review, LW and JH. Drafting the work or revising it critically for important intellectual content, C-XC and JH. All authors contributed to the article and approved the submitted version.
